# Metal Binding Ability of Small Peptides Containing Cysteine Residues

**DOI:** 10.1002/open.202000304

**Published:** 2021-04-08

**Authors:** Márton Lukács, Dóra Csilla Pálinkás, Györgyi Szunyog, Katalin Várnagy

**Affiliations:** ^1^ Department of Inorganic and Analytical Chemistry University of Debrecen Egyetem tér 1 4032 Debrecen Hungary

**Keywords:** cysteine containing peptides, metal complexes, stability constant, spectroscopic measurements, selectivity

## Abstract

The Cd(II)‐, Pb(II)‐, Ni(II)‐ and Zn(II)‐complexes of small terminally protected peptides containing CXXX, XXXC, XCCX, CX_*n*_C (*n*=1–3) sequences have been studied with potentiometric, UV/Vis and CD spectroscopic techniques. The cysteine thiolate group is the primary binding site for all studied metal ions, but the presence of a histidyl or aspartyl side chain in the molecule contributes to the stability of the complexes. For two‐cysteine containing peptides the (S^−^,S^−^) coordinated species are formed in the physiological pH range and the stability increases in the Ni(II)<Zn(II)<Pb(II)<Cd(II) order. As a conclusion, the inserting of −CXXC− sequence into the peptide makes the synthesis of peptides with high selectivity to toxic Cd(II) or Pb(II) ion possible. In addition, the spectroscopic characterization of these complexes can contribute to the discovery of the exact binding site and binding mode of longer peptides mimicking the biologically important proteins.

## Graphical abstract

**The systematic study** of small cysteine containing peptides reveals the outstanding Pb(II)‐ and Cd(II)‐binding ability of peptides with CX_*n*_C (*n*=0–3) sequence. This is well demonstrated by pM values calculated for different metal‐peptide systems at pH 7 (c_L_=0.010 mM, c_M_=0.001 mM).

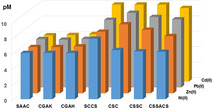

## 1 Introduction

Numerous biochemical reactions are catalysed by metalloenzymes containing cysteine thiol groups, which are the most common metal binding sites in metalloproteins and metallopeptides. Cys comprising proteins and peptides are very common not only in mammals and plants but also bacterial organisms. Their presence is related to several functions: on the one hand they, as a disulphide bridge, play a structural role in the proteins. On the other hand, they affect the maintenance of metal homeostasis. Numerous transition metals – among others copper, zinc, cadmium, manganese, cobalt, nickel, iron, molybdenium – can be coordinated by proteins containing the aforementioned residues, in which metal ions can have different functions: a structure‐stabilizing role, participation in catalysis, and functioning or regulating as cofactors.[^1^, ^2^]

Nickel is not commonly considered to be a biologically important metal, despite the fact that it is used by algae, archea, eubacteria, fungi and higher plants. This metal ion takes part in different enzymatic processes, namely hydrogen uptake and production, the conversion of carbon monoxide to carbon dioxide and urea hydrolysis; therethrough it is a catalytic cofactor of various enzyme active sites in plants and bacteria.^[3]^ Currently, the homeostasis of Ni(II) in *Helicobacter pylori* is being expansively explored.[^4^, ^5^]

Lead is a toxic metal ion, and, based on its soft character, it can bind to the proteins and it is mainly the side chain thiol groups that provide effective binding sites for it. As a consequence, this metal ion can substitute the essential metal ions (e. g. zinc(II), nickel(II)) in the metalloenzymes and metalloproteins resulting in a drastic change in their biochemical functions.[^6^, ^7^, ^8^, ^9^]

For studying the complex formation processes between metal ions and proteins, such as phytocelatins, zinc‐fingers, MTs and nickel chaperons, Cys and poly‐Cys peptides are proved to be useful models, in which the sequence concurs the coordination sites of proteins.

The complex formation processes of numerous terminally free cysteine containing di‐ and tripeptides have been studied earlier[^10^, ^11^, ^12^, ^13^, ^14^, ^15^, ^16^] among them glutathione was investigated most intensively.[^10^, ^11^, ^12^] Terminally protected peptides are, however, more suitable for the modelling of the metal binding ability of the aforementioned proteins, because the presence of the free terminal amino group significantly determines the complex formation processes.

MTs are a group of low molecular weight, cysteine‐rich intracellular proteins, which take part in various processes: maintaining intracellular metal homeostasis – by binding metals (among others, zinc and copper) – and detoxification, the oxidative stress response and cell proliferiation. More than 30 % of the sequences of these proteins are composed by cysteine, which are arranged in a series of conserved motifs: CXC, CXXC, CXCC, CCXXC and CXXCXC.[^17^, ^18^, ^19^, ^20^] Investigations were carried out with peptides that are the fragments of the native proteins and contain the aforementioned motifs in part or fully. The results reveal the formation of polynuclear Zn(II) and Cd(II) complexes with tetrathiolate coordination corresponding to the Zn(II)‐ and Cd(II)‐MT clusters.

P. Gockel et al. investigated the Zn(II) complexes of a series of tripeptides with Ac‐CXC‐NH_2_, Ac‐HXC‐NH_2_ and Ac‐CXH‐NH_2_ sequences. All these peptides form [ZnL], while the two‐cysteine containing peptides also form [Zn_2_L_2_] complexes. In latter cases the less favourable 12‐membered chelate ring sizes allow the alternative of bridging two zinc ions by Ac‐CXC‐NH_2_ peptide. The stability of (S^−^,S^−^) coordinated species are much higher than that of (S^−^,N(Im)) coordinated complexes.^[21]^ The K. Kulon et al. analysed zinc, cadmium and nickel complexes of peptides including CXC motif, as models of cluster A of acetyl coenzyme A synthase as well as MT‐3. According to their findings, the primary binding site for the metal ions is the thiol donor, and the main species formed in all cases is the [ML_2_], in which the metal ion is bound by (4S^−^) donor set. However, parallel with the increasing of pH, in the case of nickel ion, the deprotonation and coordination of amide‐NH donor groups take place, obtaining a (2S^−^,2N^−^) binding mode.^[22]^

The nickel chaperons are Cys‐ and His‐rich proteins and their metal binding abilities have been widely investigated, too. The newest studies on Helicobacter pylori's Hpn and HspA proteins state that when there is a CC motif in the sequence of peptides, nickel binds to this motif with a (2S^−^,N^−^) donor set very efficiently; the binding being more stable than Zn(II), even when histidine residues are available. The deprotonation and coordination of amide groups take place cooperatively. The results of the corresponding peptides show that in the Zn(II) and Cd(II) complexes the metal ion is coordinated by the thiol donors (2S^−^) and as expected: the formed cadmium complexes are thermodynamically more stable than the zinc ones.[^23^, ^24^]

H. Kozlowski and co‐workers studied the coordination behaviour of peptides containing CXXC motifs through the Ac‐GCASCDNCRACKK‐NH_2_, Ac‐GCASCDNCRAAKK‐NH_2_, Ac‐GCASCDNARAAKK‐NH_2_ ^[25]^ and the Ac‐ELECKDCSHVFKPNALDYGVCEKCHS‐NH_2_ peptides^[26]^ and its mutant^[27]^ in the presence of the metals listed above. These latter peptides are fragments from the loop region of HypA which normally binds Zn(II). The results reveal that only the Cys residues take part in the binding of the metal ions and Cd(II) complexes are more stable than Zn(II) complexes. In contradistinction to peptides containing CC motif the stability of the formed Zn(II) complexes is higher than that of Ni(II) complexes for all four peptides, although the binding donor set in the complexes is the same..^[24]^ Furthermore, it was concluded that the length and amino acid sequence of the linker between CXXC motifs affect the stability of the formed species.^[26]^

P. Delangle and co‐workers extended the investigation of metal binding ability of CXXC motif containing peptides (Ac‐MTCSGCRPG‐NH_2_) to other metal ions, namely Cu(I), Hg(II) and Pb(II) too.^[28]^ In these cases, the thiolate coordinated complexes are formed as well, and the stability follows the order Hg(II)>Cu(I)≫Cd(II)>Pb(II)>Zn(II), which is in accordance with what was observed for Cys‐rich ligands such as metallothioneins.

Ac‐SCHGDQGSDCSI‐NH_2_ (HS),[^29^, ^30^] Ac‐SCPGDQGSDCSI‐NH_2_ (PS),^[31]^ Ac‐SCPGDQGSDCPI‐NH_2_ ^[32]^ as well as Ac‐ACPGDDSADCPI‐NH_2_ ^[32]^ peptides were inspired by the metal binding domain of some MerR metalloregulatory proteins and were synthesized and studied by A. Jancsó and D. Szunyogh et al.. Two separated cysteines can be found in these peptides. Zn(II) and Cd(II) ions are bound to both S^−^ donor atoms, and the histidine or aspartate side chain contributes to the metal binding. The stability of these complexes is nearly as high as that of peptides containing CC, CXC or CXXC motifs.

In this manuscript, we report the results obtained in the systematic studies of cadmium(II)‐, lead(II)‐, nickel(II)‐ and zinc(II)‐complexes of small terminally protected peptides containing CXXX, XXXC, XCCX, CX_*n*_C (*n*=1–3) sequences. The stoichiometry, stability and structural characterization of the complexes of these small peptides formed in solution may provide an overview of the metal binding ability of these motifs, which can contribute to a better understanding of the structure and selectivity of native Cys‐rich proteins.

## 2 Results and Discussion

### 2.1 Acid‐Base Properties of the Ligands

The protonation constants of the ligands were determined by potentiometric titrations (the data are included in Table 1 and Table 2). It is clear from Table 1 that the basicity of the thiolate site of the one‐cysteine containing peptides displays but little variation.

**Table 1:** Protonation constants of peptides contaning one cysteinyl residue (T=298 K, I=0.2 M).

log*β*	Ac‐AAAC‐NH_2_	Ac‐SAAC‐NH_2_	Ac‐CGAA‐NH_2_	Ac‐CGAK‐NH_2_	Ac‐CGAD‐NH_2_	Ac‐CGAH‐NH_2_	Ac‐DAAC‐NH_2_^[33]^	Ac‐HAAC‐NH_2_^[34]^
[HL]	8.64(1)	8.79(1)	8.40(2)	10.38(1)	8.54(2)	8.45(2)	8.87	8.81
[H_2_L]				18.65(1)	12.68(4)	14.90(3)	12.67	15.30
p*K*(SH)	8.64	8.79	8.40	8.27	8.54	8.45	8.84	8.81
p*K*(Asp)					4.14		3.80	
p*K*(Im)						6.45		6.49
p*K*(Lys)				10.38

**Table 2:** Protonation constants of peptides contaning two cysteinyl residues (T=298 K, I=0.2 M).

log*β*	Ac‐SCCS‐NH_2_	Ac‐CSC‐NH_2_	Ac‐CGSC‐NH_2_	Ac‐CSSC‐NH_2_	Ac‐CSSACS‐NH_2_	Ac‐CC‐NH_2_^[23]^	Ac‐CCSTS‐DSHHQ‐ NH_2_^[23]^	Ac‐GSCCH‐TGNHD‐ NH_2_^[23]^	Ac‐CGC‐NH_2_^[22]^	Ac‐CPCP‐NH_2_^[22]^	Ac‐GCASC‐DNAR‐AAKK‐ NH_2_^[25]^	HS*^[29]^	PS**^[31]^
[HL]	9.00(1)	9.18(1)	9.04(1)	9.33(1)	8.82(1)	9.15			9.23	9.31			
[H_2_L]	17.02(1)	17.31(1)	17.14(1)	17.50(2)	16.91(1)	17.31			17.26	17.44			
p*K*(SH)_1_	8.02	8.13	8.10	8.17	8.09	8.16	8.21	8.20	8.03	8.13	7.97	8.32	8.30
p*K*(SH)_2_	9.00	9.18	9.04	9.33	8.82	9.16	9.07	9.23	9.23	9.31	9.37	9.19	9.09

*HS=Ac‐SCHGDQSDCSI‐NH_2_ **PS=Ac‐SCPGDQGSDCSI‐NH_2_

The deprotonation constants of −SH groups are in the 8.1–8.6 range for the peptides with N‐terminal Cys residue and 8.6–8.9 for the peptides with C‐terminal Cys residue, and these values are similar to the literature data. This means that the other side chain donor groups (Lys, Asp, His, Ser) do not affect the deprotonation process of thiol groups significantly. On the other hand, the pK values of the other side chain groups are similar to those of other peptides containing these amino acids in different environment (e. g. pK(Asp): AADAAC‐NH_2_ (3.55), ADAAAC‐NH_2_ (3.71),^[34]^ pK(Asp, His): AADAAH‐NH_2_ (3.50, 6.57), ADAAAH‐NH_2_ (3.41, 6.46)^[36]^ etc.). The higher pK(Asp) of Ac‐CGAD‐NH_2_ is probably due to the C‐terminal acid amide group, which slightly increases the basicity of the neighbouring group, as it can be seen above in the case of thiol group as well.

The formation and deprotonation constants of two‐cysteine containing peptides are collected in Table 2 completed with some literature data. The pK values fall in the 8.0–8.3 and 8.8–9.3 range, respectively, which shows that the deprotonation processes of thiol groups overlap significantly. As a consequence, the pK values cannot be unambiguously assigned to the N‐terminal and C‐terminal thiolate groups. The comparison of measured data with literature data, however, suggests that the higher pK value belongs mainly to the C‐terminal cysteinyl residue. On the other hand, there is no considerable difference in the pK values of the peptides, regardless of the distance between the two cysteine residues. The longer peptides containing CC, CXXC motifs or separated cysteines are also characterized by similar pK values (see Table 2).

### 2.2 Zinc(II), Cadmium(II) and Lead(II) Complexes of Peptides Containing one Cysteinyl Residue

The stability constants of the complexes are collected in Table S1. Mono‐ and/or bis(ligand) complexes are formed with the coordination of one or two thiolate sulphur atoms in all cases. In the protonated complexes the other side chain donor atom (histidine imidazole or lysine amino group) is protonated. Bis(ligand) complexes are present in all zinc(II)‐ and cadmium(II)‐peptide containing systems. The coordination of two thiolate sulphur atoms in the bis(ligand) cadmium(II) complexes was reinforced by UV spectroscopic parameters. The molar absorption coefficients of CdL_2_ complexes are approximately 8000–10000 M^−1^ cm^−1^ (Table 3), which is double the molar absorption coefficient of the free ligand and mono(ligand) complexes, and the intensity of the thiolate band increases in parallel with the formation of bis(ligand) complexes. The other cadmium(II) complexes coordinated by one or two cysteine residue are characterized by similar spectroscopic parameters.^[33]^ The increasing of pH results in the hydrolysis of complexes and in the formation of mixed hydroxido complexes, but the existence of hydroxido complexes cannot prevent the formation of precipitation at higher pH range.

**Table 3:** The UV/Vis parameters of cadmium(II) complexes (λ/nm, ϵ/M^−1^ cm^−1^).

		Ac‐AAAC‐NH_2_		Ac‐SAAC‐NH_2_		Ac‐CGAA‐NH_2_		Ac‐CGAK‐NH_2_		Ac‐DAAC‐NH_2_ ^[33]^		Ac‐SCCS‐NH_2_		Ac‐CSC‐NH_2_		Ac‐CSSC‐NH_2_		Ac‐CSSACS‐NH_2_
	λ	ϵ	λ	ϵ	λ	ϵ	λ	ϵ	λ	ϵ	λ	ϵ	λ	ϵ	λ	ϵ	λ	ϵ
[CdL]											209	10200	210	9300	218	9800	219	10700
[CdL_2_]	220	8300	223	13000	217	8800	217	13200	216	10000	211	27400	214	24000	223	20500	220	22100

In the cases of the lead(II) containing systems only the stability constants of mono(ligand) complexes could be determined, because the complex formation processes were accompanied by precipitation. This is probably due to the existence of polynuclear complexes with sulphur bridging. The presence of lysine side chain does not effect the complex formation processes, but the positive charge of lysine ammonium group increases the solubility of complexes in wider pH range. The formation of polynuclear complexes results in a higher number of sulphur atoms in the coordination sphere, which is in agreement with UV spectroscopic data. The formation of complexes can be examined with UV‐spectrophotometry, because these measurements were performed in ten times diluted solution and no precipitation was observed in this concentration range. The data published earlier confirm that the number of the coordinated sulphur atoms in the coordination sphere of lead(II) ion can be estimated by the λ_max_ value belongig to the S^−^→Pb(II) band.[^35^, ^36^] The deprotonation process of the thiolate groups of the ligand is accompanied by the appearance of a very intensive band around 230–240 nm. The binding of the thiolate group to the lead(II) ion, however, results in a new absorption band in the 290–330 nm range, the exact value depending on the number of thiolate groups. The series of spectra registered in Pb(II)‐peptide systems shows a significant shift of wavelength of absorption band which reflects the increasing number of bound thiolate groups (Table 4).

**Table 4:** The UV/Vis parameters of thiolate coordinated lead(II) complexes (λ/nm, ϵ/M^−1^ cm^−1^).

		Ac‐SAAC‐NH_2_		Ac‐CGAK‐NH_2_		Ac‐CGAD‐NH_2_		Ac‐SCCS‐NH_2_		Ac‐CSSC‐NH_2_		Ac‐CSSACS‐NH_2_		Ac‐Cys ^[38]^		Ac‐Pen^[38]^		Glutathione^[37]^
	λ	ϵ	λ	ϵ	λ	ϵ	λ	ϵ	λ	ϵ	λ	ϵ	λ	ϵ	λ	ϵ	λ	ϵ
1 S^−^	273	2000	276	1200	274	1650							276	2400	277	2800		
2 S^−^	314	1800			308	1000	311	3600	318	3000	318	800	309	2600	309	2600		
3 S^−^	333	2000	335	1200			338	5700	331	2000	335	1100					334	3500

The stability of different metal complexes of the peptides can be easily compared through the log*K*(M+HL) and log*K*(M+2HL) values of Ac‐CGAK‐NH_2_ and log*β*(ML) and log*β*(ML_2_) values of all other ligands (Table 5).

**Table 5:** The equilibrium constant of ML and ML_2_ complexes of one‐cysteine containing peptides (M=Zn(II), Cd(II) or Pb(II)).

log*β*	Ac‐AAAC‐NH_2_	Ac‐SAAC‐NH_2_	Ac‐CGAA‐NH_2_	Ac‐CGAK‐NH_2_	Ac‐CGAD‐NH_2_	Ac‐DAAC‐NH_2_ ^[33]^	Ac‐CGAH‐NH_2_	Ac‐HAAC‐NH_2_ ^[34]^
[ZnL]		5.00			5.3	5.32		7.02
[ZnL_2_]	9.72	9.80	9.79	8.84**	10.3	10.34	12.44	13.58
[CdL]				5.22*	6.40	6.51	6.22	7.70
[CdL_2_]	11.71	11.72	11.36	10.7**	11.6	11.9	12.62	13.34
[PbL]	6.2	6.4		5.12*			6.14	
[PbL_2_]				10.72**			11.41

*log*K*(M+HL)=log*β*(MHL)−p*K*(Lys) **log*K*(M+2HL)=log*β*(MH_2_L_2_)−2 p*K*(Lys)

The following conclusions can be drawn from these data:

(i) The position of cysteine residue in the peptide has an effect on the stability of complexes: the cysteine in the C‐termini results generally in a small stability enhancement (see Ac‐AAAC‐NH_2_ and Ac‐CGAA‐NH_2_ or Ac‐DAAC‐NH_2_ and Ac‐CGAD‐NH_2_), but the presence of a histidine residue in the other termini of the peptide causes significant a difference (see Ac‐HAAC‐NH_2_ and Ac‐CGAH‐NH_2_). These data suggest that the position of the anchor group in the C‐termini is favoured for complex formation.

(ii) The presence of another donor group in the molecule makes the bidentate coordination of the ligand possible and increases the stability in the Asp<His order.

(iii) The stability order for metal ion‐tetrapeptide complexes follows the Zn(II)<Cd(II) (≤Pb(II)) trend in all cases. The differences between the stabilities of complexes, however, depend on the other donor groups and metal ions: the presence of an aspartic acid side chain in the molecule practically does not change the difference between the stability of zinc(II) and cadmium(II) complexes, while for the histidine and cysteine containing tetrapeptides (Ac‐HAAC‐NH_2_ and Ac‐CGAH‐NH_2_) the stability of zinc(II) complexes are very close to that of cadmium(II) complexes. The latter observation reflects very well that zinc(II) favours both the histidine and cysteine binding sites, while cadmium(II) (and similarly lead(II)) prefers the coordination of thiolate group.

### 2.3 Zinc(II), Cadmium(II) and Lead(II) Complexes of Peptides Containing two Cysteinyl Residues

The peptides containing two cysteine residues form mono‐ and bis(ligand) complexes both with zinc(II) and cadmium(II), but usually only the complex with 1 : 1 stoichiometry can be detected for lead(II)‐peptide systems. In the latter case, the complex formation processes are often accompanied by precipitation. In the 1 : 1 complexes the ligands are coordinated bidentately in all cases and the stability increases in the Zn(II)<Pb(II)<Cd(II) order, which corresponds to the hard‐soft character of these metal ions. This trend is similar to that of one cysteine containing peptides, but the stability of cadmium(II) complexes is significantly higher than the stability of lead(II) complexes. The four thiolate coordinated complexes with ML_2_ stoichiometry exist in all zinc(II)‐ and cadmium(II)‐peptide systems at presence of ligand excess, and the metal ion preference follows the Zn(II)<Cd(II) order. The coordination of two or four thiolate‐S donor atoms to Cd(II) in the [ML] and [ML_2_] complexes, respectively, was confirmed by spectroscopic data (Table 3), which shows that the 2×S^−^ coordination can be characterized by 8000–11000 and 4×S^−^ coordination by 20000–27000 molar absorptivity. These parameters are in agreement with those published for CdL complex of Ac‐ELECKDCSSV‐NH_2_ (ϵ=11780 M^−1^ cm^−1^)^[27]^ and CdL_2_ complex of Ac‐ELECKDCSHV‐NH_2_ (ϵ=25670 M^−1^ cm^−1^)^[26]^ and Ac‐DYGVCEKCHS‐NH_2_ (ϵ=24900 M^−1^ cm^−1^).^[27]^

The suggested coordination mode of the Cd(II) complexes was further supported by the ^113^Cd NMR spectroscopy. In the spectrum registered in the Cd(II)‐Ac‐CSSC‐NH_2_=1 : 2 sample one peak can be observed at 648 ppm, which can be assigned to the 4 S^−^ coordinated complex (Figure S1). This parameter is similar to the ^113^Cd peaks of other Cd(II) complexes with binding of four thiolate donor atoms[^39^, ^40^] (C_2_H_5_SH: 648 ppm, n‐C_3_H_7_SH: 647 ppm, metallothioneins: 610–680 ppm).

In the cases of Pb(II)‐peptide systems the formation of polynuclear complexes can explain the appearance of precipitation. The UV/Vis spectroscopic data provide support for the existence of two and three sulphur donor atoms in the coordination sphere of lead(II) ion, but the spectroscopic parameters can be obtained only for some peptides, because the precipitation occured in the diluted solution as well. Based on these spectral data the formation of polynuclear species with thiolate bridging can be assumed. On the other hand, only the binding of three sulphur donor atoms can be detected in the PbL_2_ complex of Ac‐SCCS‐NH_2_, which, similarly to the earlier published results, suggests that lead(II) prefers the 3 S^−^ coordination mode, one ligand can bind bidentately, and the other monodentately to lead(II) ion.

The analysis of the change in the stability of [ML] and [ML_2_] complexes in the function of distance between the two cysteine residues shows that the most stable complexes exist with the peptides containing CXC and CXXC sequence, where 12‐ or 15‐membered (S^−^,S^−^) macrochelate rings are formed (Table 6).

**Table 6:** The stability constants of the mono‐ and bis(ligand) complexes of different metal ions (T=298 K, I=0.2 M).

log*β*	Ac‐SCCS‐NH_2_	Ac‐CC‐NH_2_ ^[23]^	Ac‐CSC‐NH_2_	Ac‐CGC‐NH_2_ ^[22]^	Ac‐CSSC‐NH_2_	Ac‐ELECKDCSSV‐NH_2_ ^[27]^	Ac‐CSSACS‐NH_2_	HS*[^29^, ^30^]	PS**^[31]^
[ZnL]	10.05(2)		10.11(4)		10.47(2)	*11.78*	9.16(1)	10.63	9.93
[ZnL_2_]	16.7(1)	17.63	*19.77*(5)	18.81	19.2(1)	18.12	17.07(8)	15.0	14.72
[CdL]	12.60(3)		12.69(2)		*13.04*(2)	11.88	11.47(3)	11.78	
[CdL_2_]	19.1(2)	20.71	21.0(1)	22.30	*21.1*(2)	20.77	18.6(1)	18.23	
[PbL]	10.86(4)		*12.06*(5)		11.72(3)		10.81(5)		
[PbL_2_]	15.5(2)								
member of macrochelate	9	9	12	12	15	15	18	30	30

These results are in agreement with that the formation of 12–15 membered macrocelates is also favourable for other types of ligands. For example, in the case of terminally protected multihistidine peptides, peptides containing the HXH sequence had the most favorable (N(Im), N(Im)) coordination, where a 14‐membered macrochelate ring was formed.^[41]^

**Figure 1:**
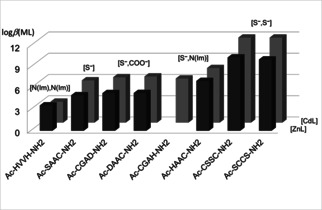
Stability constants (log*β*) of Zn(II) and Cd(II) complexes with different binding modes (data from ref. [33], [34] and [42]).

The most favoured (S^−^,S^−^) coordination for soft metal ions is well demonstrated by Figure 1, where the stability constants of Cd(II)‐ and Zn(II)‐complexes of tetrapeptides are depicted. This diagram shows that the formation of [ZnL] and [CdL] complexes with two imidazole nitrogens results in the lowest stability, and a significant enhancement of stability can be observed in the case of complexes coordinated by two thiolate groups. The substitution of one imidazole by a thiolate group results in [ZnL] and [CdL] complexes with similar stability, but the replacement of both histidyl moieties with cysteinyl residues results in a much higher enhancement of the stability of cadmium(II) complexes compared to zinc(II) complexes.

### 2.4 Nickel(II) Complexes of Peptides Containing one Cysteinyl Residue

**Figure 2:**
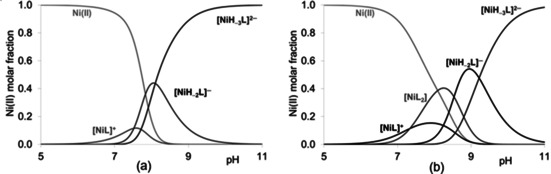
Concentration distribution curves of the species formed in Ni(II)‐AAAC 1 : 2 (a), Ni(II)‐CGAA 1 : 2 (b) systems (c_L_=1 mM).

The stability constants of Ni(II) complexes are collected in Table 7. Based on potentiometric measurements the formation of mono‐ and in some cases bis(ligand)‐complexes can be presumed. The presence of bis(ligand) complexes is not dominant in any system (Figure 2).

**Table 7:** The stability constants of nickel(II) complexes of peptides containing one cysteinyl residue (T=298 K, I=0.2 M).

log*β*	Ac‐AAAC‐NH_2_	Ac‐SAAC‐NH_2_	Ac‐DAAC‐NH_2_ ^[33]^	Ac‐HAAC‐NH_2_ ^[34]^	Ac‐CGAA‐NH_2_	Ac‐CGAK‐NH_2_	Ac‐CGAD‐NH_2_	Ac‐CGAH‐NH_2_
[NiH_2_L_2_]						28.15(6)		
[NiL_2_]				9.1	7.60(8)		7.7(1)	7.9(2)
[NiLH]				11.2		14.36(9)		
[NiL]	3.3(1)		3.81	5.17	4.09(9)		4.3(1)	4.44(7)
[NiH_−2_L]	−11.73(2)	−12.01(9)	−11.78(3)	−10.81	−12.90(4)	−11.44(1)	−12.49(4)	−11.80(4)
[NiH_−3_L]	−19.83(2)	−19.36(2)	−20.60(4)	−21.53	−22.11(4)	−22.24(5)	−21.55(5)	−20.77(6)
p*K*(amide)_12_	7.52		7.80	7.99	8.38		8.50	8.12
p*K*(amide)_3_	8.10	7.35	8.82	10.72	9.06		9.21	8.97
p*K*(amide)av.	7.71		8.14	8.90	8.73	8.60	8.60	8.40

The existence of protonated complexes of Ac‐CGAK‐NH_2_ ([NiHL], [NiH_2_L_2_]) is due to the protonated lysyl side chain in the complexes. Above pH 7 new base consuming processes can be observed, which correspond to the deprotonation and coordination of peptide amide groups. The nickel(II) ion induced deprotonation of amide nitrogens takes place in a cooperative manner, which means that the deprotonation of the first and the second amide group occurs in one step and this process can be characterized by p*K*(amid)_1,2_=(log*β*([ML])‐(log*β*([MH_−2_L]))/2. For comparison, the average values of p*K*(amid) are also calculated (p*K*(amid)av=(log*β*([ML])‐(log*β*([MH_−3_L])/3 or (log*β*([MHL])‐(log*β*([MH_−2_L])/3 for Ac‐CGAK‐NH_2_). (Table 7, last three rows).

The stability of NiL complexes increases, if another donor atom is present in the molecules and the order of stability is the same as for the other three studied metal ions:

$$\mathrm{Ac}\text{-}\mathrm{AAAC}\text{-}\mathrm{NH}{}_{2}<\mathrm{Ac}\text{-}\mathrm{DAAC}\text{-}\mathrm{NH}{}_{2}<\mathrm{Ac}\text{-}\mathrm{HAAC}\text{-}\mathrm{NH}{}_{2}$$

$$\mathrm{Ac}\text{-}\mathrm{CGAA}\text{-}\mathrm{NH}{}_{2}<\mathrm{Ac}\text{-}\mathrm{CGAD}\text{-}\mathrm{NH}{}_{2}<\mathrm{CGAH}\text{-}\mathrm{NH}{}_{2}$$

The thiolate coordination is supported by the appearance of a S^−^→Ni(II) CT band in the UV/Vis spectra around 300–330 nm with a high intensity (ϵ∼500–1000 M^−1^ cm^−1^, Table 8).

**Table 8:** The UV/Vis parameters of nickel(II) complexes of the studies peptides **(**λ_max_/nm, ϵ/M^−1^ cm^−1^).

	Ac‐AAAC‐NH_2_	Ac‐CGAA‐NH_2_	Ac‐CGAK‐NH_2_	Ac‐CGAD‐NH_2_	Ac‐CGAH‐NH_2_
pH∼7.5					
λ_max_ (ϵ)	335 (1410)	335 (516)		333 (525)	336 (843)
pH∼11, [NiH_−3_L]					
λ_max_ (ϵ)	443 (216)	434 (172)	443 (126)	433 (122)	436 (200)
λ_max_ (ϵ) (sh)	530 (66)	512 (98)	525 (61)	511 (63)	519 (105)

The enhancement of the stability of Ac‐CGAD‐NH_2_, Ac‐CGAH‐NH_2_ and Ac‐DAAC‐NH_2_,^[33]^ Ac‐HAAC‐NH_2_ ^[34]^ is caused by the bidentate coordination of the ligands with cysteine in either the N‐terminal or C‐terminal position.

The presence of a histidyl residue in the tetrapeptide containing N‐terminal cysteine, however, has a much smaller effect than the reverse layout: log*β*([NiL])=3.81 and 5.17 for Ac‐DAAC‐NH_2_ and Ac‐HAAC‐NH_2_, while 4.3 and 4.44 for Ac‐CGAD‐NH_2_ and Ac‐CGAH‐NH_2_.

**Figure 3:**
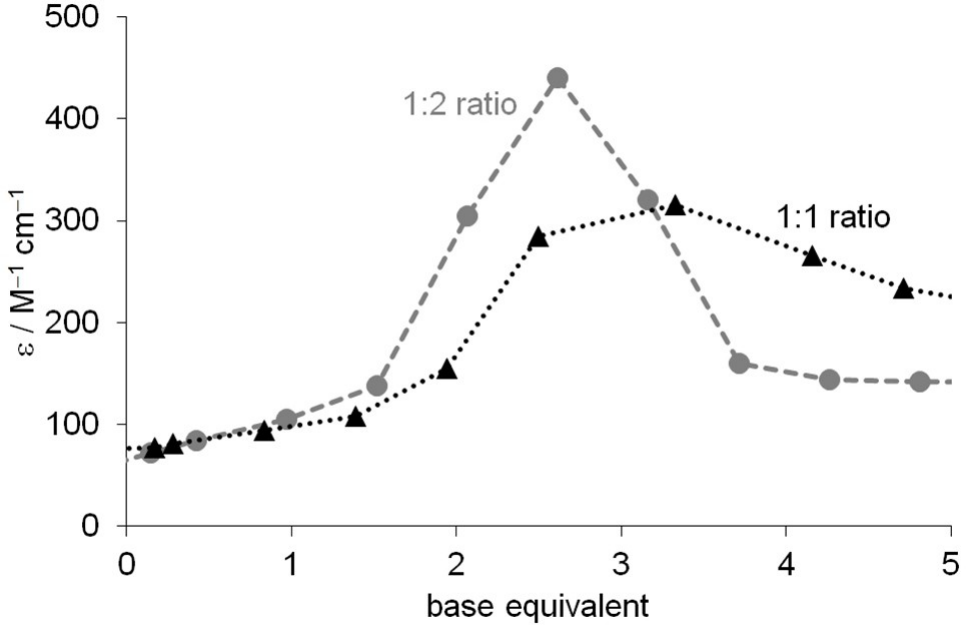
Change of the molar absorptivity of Ni(II):Ac‐CGAH‐NH_2_ 1 : 1 and 1 : 2 solutions at 408 nm in the function of equivalent of base (c_L_=1 mM).

The stoichiometries of complexes calculated from the potentiometric data are 1 : 1 and 1 : 2. The octahedral geometry of the complexes, however, would make the coordination of more donor groups possible. It suggests that the 1 : 1 or 1 : 2 complexes are not mononuclear species but polynuclear ones with thiolate bridging ([(NiL)_x_] and [(NiL_2_)_x_]). This assumption is confirmed by the change of the intensity of the band around 400–430 nm, which follows maximum curves in the function of equivalent of base (Figure 3), and this maximum is shifted to a smaller equivalent for Ni(II)‐ligand 1 : 2 ratio. This indicates a substantial amount of thiolate bridging, which is disrupted by the additional base.^[41]^

The formation of [NiH_−2_L] and [NiH_−3_L] complexes is accompanied by a new band around 440 nm with a much smaller intensity (ϵ∼200–300 M^−1^ cm^−1^, Table 8), which corresponds to the ((N^−^)_x_,S^−^) (x=2,3) or (N^−^,N^−^,S^−^,N(Im)/COO^−^) coordinated nickel(II) complexes with square planar geometry. These bands, however, are not sharp peaks, a shoulder can be observed around 470–490 nm proving the presence of thiolate S or histidine imidazole in the coordination sphere of the metal ion.

The exact coordination mode of the [NiH_−2_L] and [NiH_−3_L] complexes can be concluded from the analysis of CD spectra. In all cases the amide nitrogen coordinated species are CD active and the shape of the registered CD spectra does not change, only its Cotton effects increase with the increasing of pH.

**Figure 4:**
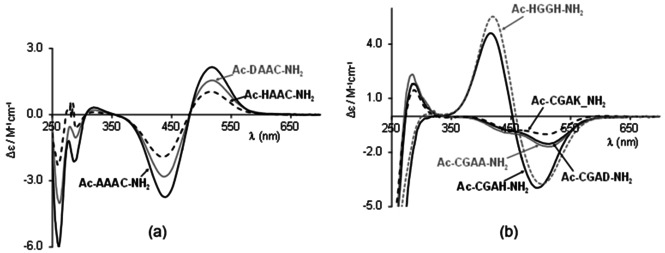
The CD spectra of [NiH_−3_L] complexes of different tetrapeptides containing cysteinyl residue in the C‐termini (a) and N‐termini (b).

Figure 4 shows the CD spectra of [NiH_−3_L] complexes of different types of tetrapeptides. These spectra obviously display the difference between the coordination mode of different types of tetrapeptides and based on the CD spectra (and coordination mode) the studied peptides can be divided into three groups:

$$\left(i\right)\;\mathrm{Group}\;I:\;\mathrm{Ac}\text{-}\mathrm{YXXC}\text{-}\mathrm{NH}{}_{2},\;Y=A,\;S,\;D\;\mathrm{or}\;H$$

**Scheme 1:**
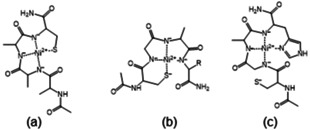
The schematic structures of [NiH_−3_L] complexes of tetrapeptides: Ac‐AAAC‐NH_2_ (a), Ac‐CGAX‐NH_2_, X=A, D or K (b) and Ac‐CGAH‐NH_2_ (c).

There is a positive Cotton effect at 517–518 nm and negative Cotton effect at 438 nm in the CD spectra (Figure 4a). These spectra support the claim that the C‐terminal cysteinyl thiolate group is the primary anchor and the two or three preceding deprotonated amide nitrogens are bound to nickel(II) ion forming (5,6) or (5,5,6)‐membered joined chelates (Scheme 1a). The binding of carboxylate or imidazole group in the [NiH_−2_L] complex hinders the deprotonation of amide nitrogen, and the pK value of the third amide nitrogen increases in the Ac‐AAAC‐NH_2_<Ac‐DAAC‐NH_2_<Ac‐HAAC‐NH_2_ order.

(ii) Group II: Ac‐CGXY‐NH_2_, Y=A, K or D.

The CD spectra are different from those of peptide group I (two negative Cotton effect at 517 nm and 450 nm, Figure 4b). The primary anchor group is the N‐terminal cysteinyl thiolate group and the neighbouring two or three deprotonated amide nitrogens take part in the nickel(II) binding forming (5,5) or (5,5,5)‐membered joined chelates (Scheme 1b). The weak binding of C‐terminal aspartyl carboxylate group cannot be excluded, but it does not affect the deprotonation of the third amide nitrogen.

$$\left(\mathrm{iii}\right)\;\mathrm{Group}\;\mathrm{III}:\;\mathrm{Ac}\text{-}\mathrm{CGAH}\text{-}\mathrm{NH}{}_{2}:$$

The CD spectra of the nickel(II) complex of this peptide differs from both the spectra of nickel(II) complexes of group I and group II, but is very similar to that of Ac‐HGGH‐NH_2_. This fact indicates that both cysteinyl and histidyl residues bind the nickel(II) ion in the first step, but the deprotonation and coordination of amide nitrogens “starts” from the C‐terminal part of the molecule resulting in (S^−^,N^−^,N^−^,N(Im)) coordination mode for [NiH_−2_L] and (N^−^,N^−^,N^−^,N(Im)) for [NiH_−3_L]. In both complexes (5,5,6)‐membered joined chelates are formed (Scheme 1c) and the deprotonation of the third amide nitrogen is less hindered than either Ac‐HAAC‐NH_2_ or Ac‐CGAD‐NH_2_.

In conclusion, we can state that cysteine is usually the primary binding site for nickel(II) ion to induce the deprotonation and coordination of amide nitrogen regardless of whether the cysteine is on the C‐termini or N‐termini. The presence of a histidyl residue in the C‐termini, however, changes the nickel(II) binding preference, which means that nickel(II) prefers binding on the C‐terminal part of the molecule to binding to the cysteinyl residue.

### 2.5 Nickel(II) Complexes of Peptides Containing two Cysteinyl Residues

**Scheme 2:**
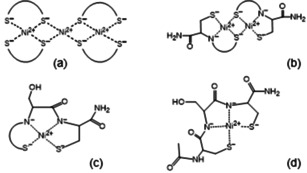
The schematic structures of [Ni_3_L_4_] **(a)**, [(Ni_2_H_−2_L_2_])_x_] **(b)**, [NiH_−2_L] **(c)** and [NiH_−3_L] **(d)** complexes.

The stoichiometry and stability of complexes are collected in Table 9. The data show that mono‐ and polynuclear‐complexes are formed. The [NiL] and [Ni_3_L_4_] complexes are exclusively thiolate coordinated species. The ligand is bound bidentately in NiL complex and the formation of 15‐membered macrochelate with −CXXC− sequence is the most favourable for nickel(II) complexes, similarly to cadmium(II) and zinc(II) complexes. Polynuclear [Ni_3_L_4_] complexes can be detected in all cases, in which thiolate behaves as a bridge (Scheme 2a).

**Table 9:** The stability constants of nickel(II) complexes of peptides containing two cysteinyl residues (T=298 K, I=0.2 M).

	Ac‐SCCS‐NH_2_	Ac‐CSC‐NH_2_	Ac‐CSSC‐NH_2_	Ac‐CGSC‐NH_2_	Ac‐CSSACS‐NH_2_
[Ni_3_L_4_]	43.93(8)	42.64(8)	39.62(9)	38.0(1)	36.3(2)
[NiL]			8.34(3)	7.90(3)	7.58(6)
[NiH_−1_L]			−0.06(4)	0.38(8)	
[(Ni_2_H_−2_L_2_)_x_]	11.55(2)	8.89(8)			
[(Ni_2_H_−3_L_2_)_x_]	0.98(6)				
[NiH_−2_L]		−6.38(6)	−10.04(4)	−8.93(5)	−9.48(4)
[NiH_−3_L]			−21.05(4)	−19.98(5)	−20.61(6)
p*K*(amide)_1_	5.3*	6.3*	8.40	7.52	8.53
p*K*(amide)_2_	10.57	9.2*	9.98	9.31	
p*K*(amide)_3_			11.01	11.05	11.13

*estimated value on the base of distribution curves

The coordination of the S^−^ group is accompanied by the appearance of an intensive band around 400–435 nm, and as for one cysteine containing peptides, the molar absorptivity follows maximum curves in the function of equivalent of base (Figure S2), and this maximum is shifted to a smaller equivalent for Ni(II)‐ligand 1 : 3 ratio.

The increase of pH results in extra base consuming processes corresponding to the deprotonation and coordination of amide nitrogens. The stoichiometry of complexes and the calculated or estimated pK(amide) values clearly show that for Ac‐SCCS‐NH_2_ and Ac‐CSC‐NH_2_ peptides, two amide nitrogens are deprotonated and these processes take place in two separate steps. If two or three amino acids are between the two cysteines, the deprotonation of the first and second amide nitrogens occurs in a pH range close to each other (Ac‐CSSC‐NH_2_, Ac‐CGSC‐NH_2_) or in cooperative steps (Ac‐CSSACS‐NH_2_) (see Table 9, last three rows).

**Figure 5:**
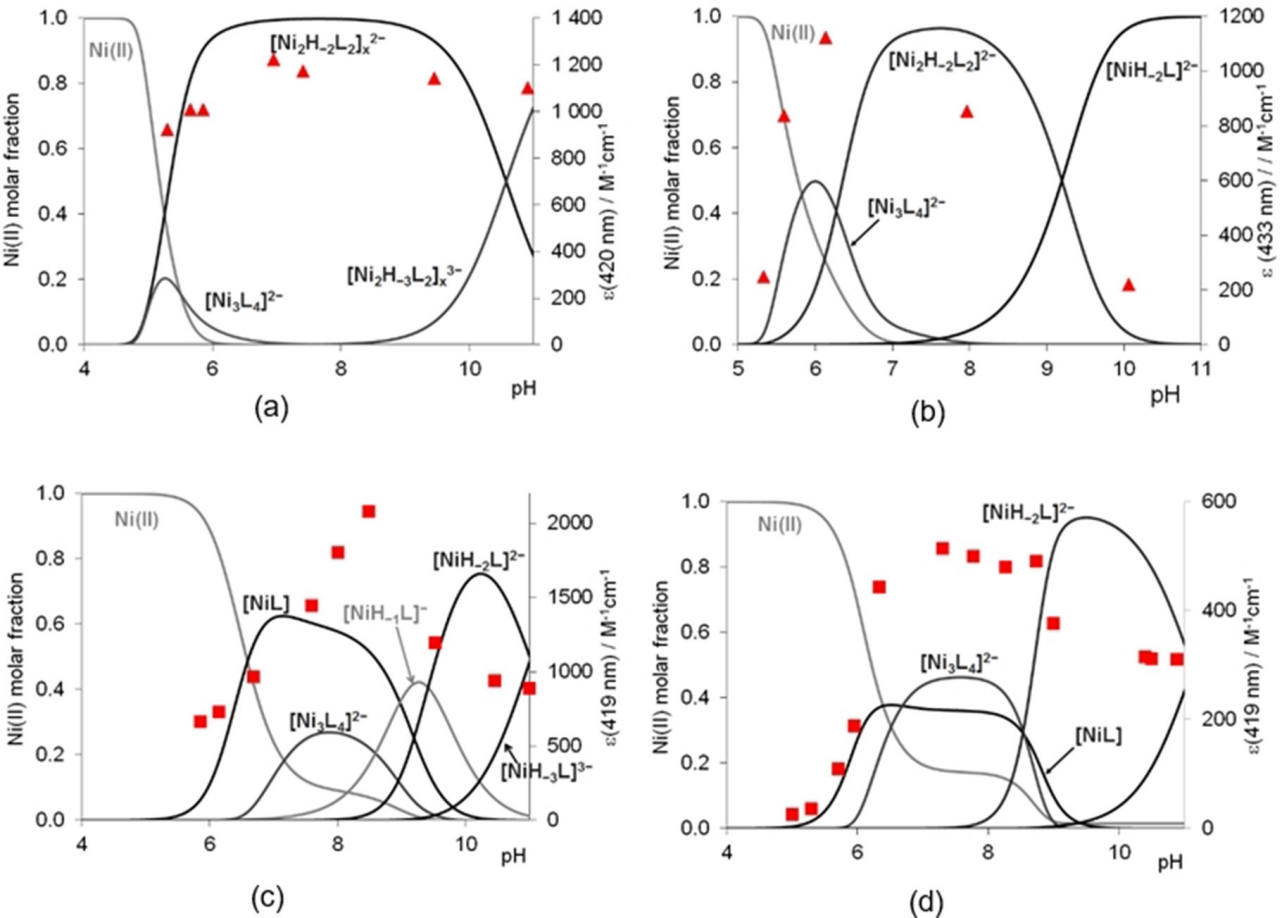
Concentration distribution curves of equimolar solution of Ni(II):Ac‐SCCS‐NH_2_ (a), Ni(II):Ac‐CSC‐NH_2_ (b), Ni(II):Ac‐CSSC‐NH_2_ (c) and Ni(II):Ac‐CSSACS‐NH_2_ (d) systems and the absorption values at 419–433 nm in function of pH (c_L_=1 mM).

Both the stoichiometry and the structure of amide coordinated species are different and these depend on the distance between cysteine residues in the molecule. These differences are well demonstrated by the distribution curves of the equimolar systems of different peptides, where the change of the molar absorptivity of the band at 400–435 nm are also depicted in the function of pH (Figure 5.).

The deprotonation of one amide nitrogen results in square planar geometry with (S^−^,N^−^,S^−^) coordination mode, (5,5) joined chelate for Ac‐SCCS‐NH_2_, and 5‐membered chelate joined a macrochelate for Ac‐CSC‐NH_2_, Ac‐CSSC‐NH_2_/Ac‐CGSC‐NH_2_ and Ac‐CSSACS‐NH_2_ peptides. This tridentate binding mode, however, does not saturate the coordination sphere of nickel(II) ion, and the saturation of the fourth position proceeds differently in the case of different ligands. For complexes of Ac‐SCCS‐NH_2_ and Ac‐CSC‐NH_2_ peptides, the thiolate bridged di‐ or polynuclear structure exists at higher pH, where the coordinated thiolate is able to bind as a bridge to another nickel(II) ion, forming [(Ni_2_H_−2_L_2_)_x_] complexes (Scheme 2b), and this complex predominates in wide pH range (Figure 5a and b). The increasing size of macrochelate for Ac‐CSSC‐NH_2_/Ac‐CGSC‐NH_2_ and Ac‐CSSACS‐NH_2_ prevents sterically the formation of polynuclear complexes. On the other hand, it promotes the deprotonation and coordination of the second amide nitrogen. The formation of mononuclear [NiH_−2_L] species with

(S^−^,N^−^,N^−,^S^−^) donor set becomes (Scheme 2c) more favourable if the distance between cysteinyl residues increases. Figure 5c and d declare this trend well, the ratio of (S^−^,N^−^,S^−^) coordinated species is low in the Ni(II)‐Ac‐CSSC‐NH_2_ system, while the cooperative deprotonation of two amide nitrogens can be observed for Ni(II)‐Ac‐CSSACS‐NH_2_ system.

The deprotonation and coordination of the second amide nitrogen also take place in the Ni(II)‐Ac‐CSC‐NH_2_‐system.

[NiH_−2_L] complex is favoured for nickel(II) ion, because in the species bound by (S^−^,N^−^,N^−^,S^−^) donor set (5,5,6)‐membered joined chelates (Scheme 2d) are present, saturating the coordination sphere of the metal ion. The break of polynuclear [(Ni_2_H_−2_L_2_)_x_] structures, however, slightly prevents this process.

The polynuclear structure is the most favourable for Ac‐SCCS‐NH_2_ ligand, and the deprotonation of another amide nitrogen takes place only above pH 10, while one thiolate donor atom is displaced from the coordination sphere forming [(Ni_2_H_−3_L_2_)_x_] complex.

If one or more amino acids are present between the two cysteinyl residues, the [NiH_−2_L] complex predominates in the strong alkali solution (above pH 9), and the deprotonation and coordination of the third amide nitrogen can be observed only above pH 11 (Figure 5c and d). The binding of two thiolates and two amide nitrogens hinders the next deprotonation step significantly, the p*K*_3_(amide) values are ∼11, in contrast with one cysteine containing peptides, where the [NiH_−3_L] complexes predominate above pH 9.

The UV/Vis and CD spectroscopic measurements provide good evidence for these complex formation processes. The change of characteristic Ni^2+^→S^−^ band around 400–435 nm in the function of pH (Figure 5) shows that the complexes of Ni(II)‐Ac‐SCCS‐NH_2_ systems have polynuclear structures in the whole pH range, while the formation of mononuclear [NiH_−2_L] species follows the [Ni_3_L_4_] and [Ni_2_H_−2_L_2_] complexes if the distance between two cysteines increases and it is accompanied by a decrease of intensity of Ni^2+^→S^−^ band. The comparison of UV/Vis spectra detected in strong alkaline solution (Figure S3) reinforces the presence of complexes with different structures.

**Figure 6:**
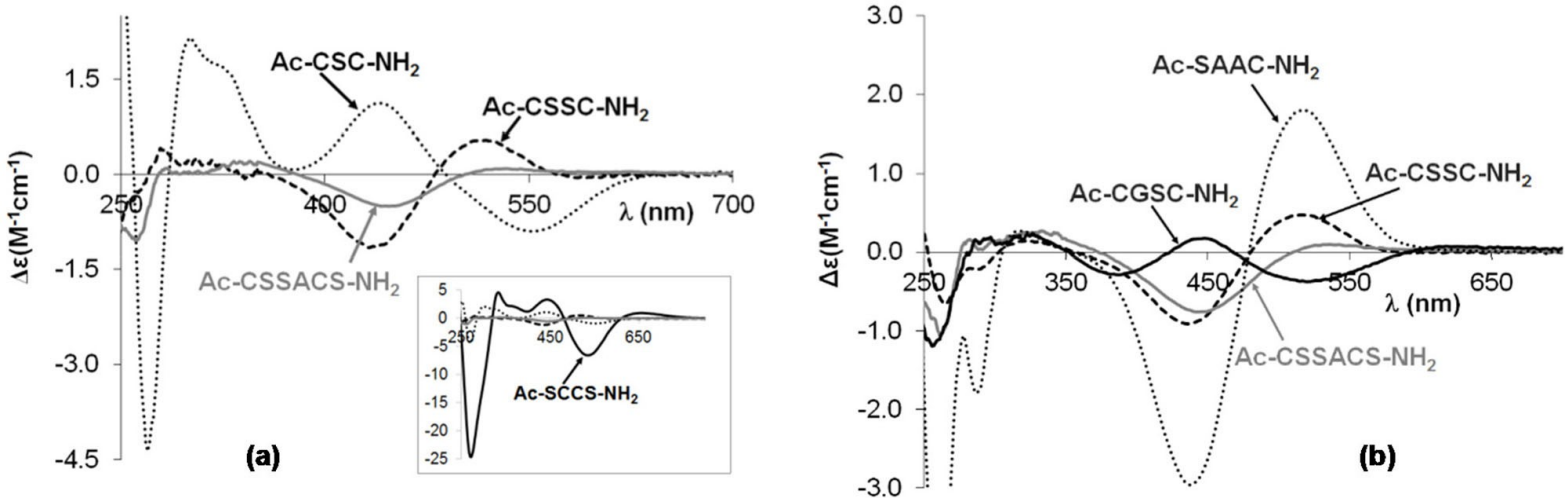
CD spectra of [NiH_−2_L]/ [(Ni_2_H_−3_L_2_)_x_] **(a)** and [NiH_−3_L] **(b)** complexes of peptides containing two cysteinyl residues.

A similar conclusion can be drawn from CD spectra. The distinct change of the spectra registered in the Ni(II)‐Ac‐CSC‐NH_2_ and Ni(II)‐Ac‐CSSC‐NH_2_ systems can be seen in Figure S4, confirming the different structure of amide nitrogen coordinated species. This difference is much clearer if the CD spectra characteristic for [NiH_−2_L] ([Ni_2_H_−3_L_2_)_x_]) and [NiH_−3_L] species are compared (Figure 6). The spectra of Ni(II)‐Ac‐SCCS‐NH_2_ and Ni(II)‐Ac‐CSC‐NH_2_ complexes are totally different from each other and from those of Ni(II)‐Ac‐CSSC‐NH_2_ and Ni(II)‐Ac‐CSSACS‐NH_2_ (Figure 6a). These spectra correspond to the formation of polynuclear species in Ni(II)‐Ac‐SCCS‐NH_2_ system, and stable [NiH_−2_L] complex of Ac‐CSC‐NH_2_ with favourable (6,5,5)‐membered joined chelates. The CD spectra of [NiH_−2_L] complex of Ac‐CSSC‐NH_2_ and Ac‐CSSACS‐NH_2_ are similar to each other which is characteristic of the complex with (5,5)‐chelates joined with a 9‐or 12‐membered macrochelate. This macrochelate is evolved with the binding of the distant thiolate sulphur atom.

On the other hand, the comparison of CD spectra of [NiH_−3_L] complexes of one and two cysteine containing peptides (Figure 6b) verifies that the C‐terminal cysteine is the primary binding site, and the C‐terminal thiolate and preceding amide nitrogen donor atoms coordinate the nickel(II) ion (Scheme 2c and d). This fact is obvious from the similarity of the CD spectrum registered in the Ni(II)‐Ac‐SAAC‐NH_2_, ‐Ac‐AAAC‐NH_2_, ‐Ac‐CSSC‐NH_2_ and Ac‐CSSACS‐NH_2_ system. Furthermore, the spectrum of Ni(II)‐Ac‐CGSC‐NH_2_ complex shows inversion, and this observation is in agreement with those previous findings for protected histidine containing peptides^,[44,45]^ according to which mirror images occur in the CD spectra if glycine occupies the third position with respect to the coordinated histidine residue.

## 3 Conclusions

The systematic studies of cadmium(II), lead(II), zinc(II) and nickel(II) complexes of one‐ and two‐cysteine containing oligopeptides were performed (i) to determine the different coordination mode of complexes formed with peptides containing one or two cysteines in different positions (ii) to determine the characteristic spectral parameters of the complexes with different coordination mode (iii) to study the effects of other donor groups in the molecule and the distance between the two cysteinyl residues, respectively, on the complex formation processes.

The results prove that the cysteine thiolate group of one‐cysteine containing peptides is the primary binding site for cadmium(II), lead(II) and zinc(II) ions, but the presence of a histidyl or aspartyl side chain in the molecule contributes to the stability of the complexes. Although the stability of cadmium(II) complexes is the highest, the presence of both cysteine and histidine in the ligand significantly increases the stability of zinc(II) complexes.

Nickel(II) ion can induce the deprotonation of peptide amide nitrogens regardless of whether the cysteine is in the C‐terminal or N‐terminal position. If both cysteine and histidine are present in the molecule (Ac‐HAAC‐NH_2_ ^[34]^ or Ac‐CGAH‐NH_2_), the C‐terminal donor group is always the primary anchor group, and the N‐terminal donor group is replaced by the third amide nitrogen in the [NiH_−3_L] species, which means that these two side chain groups are practically equivalent with respect to nickel(II) ion, and their positions determine the coordination modes.

**Figure 7:**
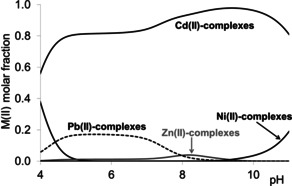
Concentration distribution curves of model system containing Cd(II) : Pb(II) : Zn(II) : Ni(II) : Ac‐CSSC‐NH_2_ in 1 : 1 : 1 : 1 : 1 ratio (c_L_=1 mM).

For two‐cysteine containing peptides the bidentate coordination via two sulfur atoms of the peptide is characteristic of all metal ions in the physiological pH range, and this coordination mode is the most favourable for cadmium(II). The cadmium(II) selectivity of these ligands is well demonstrated by Figure 7, where the calculated distribution curves of Cd(II):Pb(II):Zn(II):Ni(II):Ac‐CSSC‐NH_2_=1 : 1 : 1 : 1 : 1 are depicted.

The coordinated thiolate group, however, can behave as a bridging ligand resulting in complexes with polynuclear structures. The formation of polynuclear complexes can be detected for nickel(II) ion, and the closer the two cysteines are in the peptide, the more prone it is to form polynuclear species. In the case of Ac‐SCCS‐NH_2_ peptide polynuclear species with thiolate bridging exist in the whole pH range. With increasing the distance between the cysteinyl residues the dominance of mononuclear [NiH_−2_L] complex increases with (S^−^,N^−^,N^−^,S^−^) coordination mode. This structure stabilizes the complexes and shifts the deprotonation and coordination of the third amide nitrogen above pH 11.

Based on these observations, we can conclude that:

(i) The inserting of −CXXC− sequence into the peptide makes the synthesis of peptides with high selectivity to toxic cadmium(II) or lead(II) ion possible.

(ii) The formation of polynuclear nickel(II) complexes should be taken into account in the nickel(II)‐multicysteine peptide containing system, although the literature data published for similar peptides contain only a simplified model with mononuclear species. On the other hand, it is worth emphasising that the ratio of polynuclear species is significant in the concentration ranges used in potentiometric and spectroscopic studies. The decreasing of concentration of nickel(II) ion and ligand by several orders of magnitude (e. g.10^−5^–10^−6^ M) results in a negligible amount of thiolate bridged polynuclear complexes.

(iii) In addition, the spectroscopic characterization of the complexes of these simple peptides with different coordination mode can contribute to the discovery of the exact binding site and binding mode of longer peptides mimicking the biologically important proteins.

## Experimental Section

### Material and Methods

Chemicals and solvents used for synthetic purposes were provided from commercial sources in the highest available purity and used without further purification. The Rink Amide AM resin (substituation: 0,69 mmol/g), all of the N‐flourenylmethoxycarbonyl (Fmoc)‐protected amino acids (Fmoc‐Ala‐OH, Fmoc‐Asp(OtBu)‐OH, Fmoc‐Cys(Trt)‐OH, Fmoc‐Gly‐OH, Fmoc‐Hys(Trt)‐OH, Fmoc‐Lys(Boc)‐OH, Fmoc‐Ser(Trt)‐OH) and 2‐(1‐H‐benzotriazole‐1‐yl)‐1,1,3,3‐tetramethyluronium tetrafluoroborate (TBTU) are Novabiochem (Switzerland) products. Ac‐CGAK‐NH_2_ peptide (purity more than 95 %) was purchased from Synpeptide Co. Ltd. 2‐methyl‐2‐butanol, N‐hydroxybenzotriazole hydrate (HOBt⋅H_2_O), N‐methyl‐pyrrolidone (NMP), 2,2’‐(ethylenedioxy)diethanethiol (DODT) and triisopropylsilane (TIS) were received from Sigma‐Aldrich Co., while trifluoroacetic acid (TFA) and N,N‐diisopropyl‐ethylamine (DIPEA) were Merck Millipore Co. products. Peptide‐synthesis grade acetic anhydride (Ac_2_O) and N,N‐dimethylformamide (DMF) were bought from VWR International, as piperidine, dichloromethane (DCM), diethyl ether (Et_2_O), acetic acid (AcOH) and acetonitrile (ACN) from Molar Chemicals Ltd.

KOH and KCl, used during pH‐potentiometric titrations, were purchased from Merck.

Lead(II)‐trifluoracetate (Pb(CF_3_COO)_2_) solution was prepared from Pb(OH)_2_ and TFA. The Pb(OH)_2_ was obtained from reaction of lead(II) nitrate with ammonia solution. The exact acid content of solution was determined by potentiometric titration using Gran method.^[46]^ The stock solutions of other metal ions were acquired from analytical grade reagents (Pb(NO_3_)_2_, Cd(NO_3_)_2_, Ni(NO_3_)_2_, Zn(NO_3_)_2_, NiCl_2_ and ZnCl_2_) and their concentrations were determined gravimetrically via the precipitation of oxinates and/or pH‐potentiometrically using EDTA as chelating agent and/or using AAS method.

The concentrations of the peptide solution was determined in each case via pH‐potentiometric titrations.

### Peptide Synthesis

Excluding Ac‐CGAK‐NH_2_, which was purchased from Synpeptide Co., Ltd, all of the peptides were synthesized by solid phase peptide synthesis performed using a microwave‐assisted Liberty 1 Peptide Synthesizer (CEM, Matthews, NC). Fmoc protected amino acid derivatives were introduced according to the Fmoc/tBtu technique and the TBTU/HOBt/DIPEA activation strategy. Cleaving on the α‐amino protecting group of amino acids and resin was performed by 30 W microwave power for 180 s at 80 °C carrying out with 20 V/V % piperidine and 0.1 M HOBt in DMF. Four time excess of amino acids and 30 W microwave power for 300 s were used for coupling at 80 °C in the presence of 0.5 M HOBt and 0.5 M TBTU in DMF as activator and 2 M DIPEA in NMP as activator base. The free amino terminus was treated with DMF containing 5 V/V % Ac_2_O and 6 V/V % DIPEA to produce the acetylated amino group. After setting up the peptide sequence, peptides were cleaved from their respective resins, with the simultaneous removal of the side chain protective groups, with aid of a mixture of TFA/TIS/H_2_O/DODT in 94/2.5/2.5/1 V/V % for 1.5–2 h at room temperature. The solutions of the free peptides were separated from the resin by filtration in each case. The crude peptides were regained from the solution in question by precipitation with cold diethyl ether. The precipitate was washed and separated from it, then dried and re‐dissolved in water, finally frozen for lyophilisation.

**Scheme 3:**
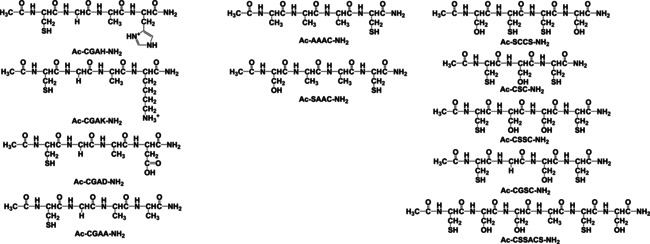
Structural formulae of the studied peptides.

The purity of the synthesized peptides was checked by analytical RP‐HPLC using a Jasco instrument equipped with a Jasco MD 2010 plus wavelength detector monitoring the absorbance at 222 nm that is characteristic of the peptide bond. The chromatographic conditions were the following: column: Europa peptide C18 (205×4.6 mm, 120 Å pore size, 5 μm particle size); elution: gradient elution was performed using a solvent a (0.1 V/V % TFA in water) and solvent B (0.1 V/V % TFA in acetonitrile) at a flow rate of 1 mL min^−1^. The purity was also checked by mass spectrometry, moreover, the pH‐potentiometric titrations of the ligands also confirmed the identity and purity. The schematic structures of the studied peptides are shown in Scheme 3.

### Potentiometric Measurements

pH‐potenciometric titrations were performed on 3.00 mL samples at 1–2 mM total ligand concentration with the use of carbonate‐free stock solution (∼0.2 M) of potassium hydroxide. The metal to ligand ratios were selected between 1 : 1 and 1 : 4. During the measurements argon was bubbled through the samples to ensure the absence of oxygen and carbon dioxide. The sample solutions were stirred by a VELP Scientific magnetic stirrer. All pH‐potentiometric measurements were performed at 298 K. The ionic strength was adjusted to 0.2 M with KCl in the case of nickel(II) and zinc(II) complexes, while KNO_3_ was used for cadmium(II) and lead(II) to avoid the formation of chlorido complexes. During measurements two types of instruments were used: a MOLSPIN pH‐meter equipped with a 6.0234.100 and 6.0234.110 combination glass electrodes (Metrohm) and the titrant was dosed by means of a MOL‐ACS burette controlled by a computer (in case of nickel(II), zinc(II) and cadmium(II) complexes). A computer controlled METTLER TOLEDO titrator equipped with a 6.0255.100 combined glass electrode (Metrohm) was used in the case of lead(II) complexes. The recorded pH readings were converted to hydrogen ion concentration as described by Irving at al.^[47]^ The equilibrium conditions were applied: the system is in equilibrium if the maximum change of pH is 0.001 unit for 30 s. The protonation constants of the ligands and the overall stability constants (lg *β_pqrs_)* of the metal complexes were calculated by means of the general computational programs: SUPERQUAD^[48]^ and PSEQUAD.^[49]^

(1) $$p\;M+q\;H+r\;L\overset{⇀}{↽}M{}_{p}H{}_{q}L{}_{r}$$

(2) $$\beta_{pqr}=\frac{{[M}_{p}H_{q}L_{r}]}{{\left[M\right]}^{p}{\left[H\right]}^{q}{\left[L\right]}^{r}}$$

The hydrolysis models of the metal ions had been determined in previous works. The following stability constants (log*β*) were calculated: [Pb(OH)]^+^ (log*β=*−7,32), [Pb_4_(OH)_4_]^4+^ (log*β=*−19,98) [Pb_6_(OH)_8_]^4+^ (log*β=*−42,62),^[50]^ [Zn(OH)_2_] (log*β=*−16,55), [Zn(OH)_4_]^2−^ (log*β=*−39,71),^[51]^ [Cd(OH)]^+^ (log*β=*−9,60).^[52]^

### Spectroscopic Studies

UV/Vis, CD, ^1^H and ^113^Cd NMR spectra were recorded to gain more information about the structure of the complexes formed in the investigated systems.

UV/Vis spectra of the nickel(II) complexes were registered from 250 to 900 nm on a PerkinElmer Lambda 25 scanning spectrophotometer in the same concentration range as that used for pH‐potentiometry. Circular dichroism spectra of nickel(II) complexes were also recorded on a Jasco‐810 spectropolarimeter applied 1 mm and/or cm cells in the 220–800 nm wavelength range. Nuclear magnetic resonance (^1^H NMR) spectra were recorded on a Bruker AM 400/360 MHz FT‐NMR spectrometer. The chemical shifts were referenced to internal sodium 3‐(trimethyl‐silyl)‐1‐propane sulfonate (TSP, δ_TSP_=0 ppm) and D_2_O was used as a solvent. DCl/DNO_3_ and KOD/NaOD were used to set the pH of the samples. The total ligand concentration was 20 mM in the NMR experiments. ^113^Cd NMR spectra were recorded in DMSO‐d^6^ solution. The spectra were externally referenced to 0.1 M Cd(NO_3_)_2_ in DMSO‐d^6^. Acquisition parameters were the following: pulse length 15 μs (60°), pulse repetition time 10 s, spectral width 42 kHz, data point 65536 and total number of collection scans 16000. The spectra were evaluated by MestreNova software. Checking of the purity of the peptides was performed by a Bruker micrOTOF‐Q 9 ESI‐TOF instrument.

## Conflict of interest

The authors declare no conflict of interest.

## Supporting information

As a service to our authors and readers, this journal provides supporting information supplied by the authors. Such materials are peer reviewed and may be re‐organized for online delivery, but are not copy‐edited or typeset. Technical support issues arising from supporting information (other than missing files) should be addressed to the authors.

Supplementary
