# Supplementary material for: Metal Binding Ability of Small Peptides Containing Cysteine Residues
Source: ChemistryOpen. 2021 Apr 8;10(4):451–63. doi: 10.1002/open.202000304 (PMC8028610; doi:10.1002/open.202000304)
Supplement: Supplementary file 1 — Supplementary [file OPEN-10-451-s001.pdf]

ChemistryOpen

Supporting Information

Metal Binding Ability of Small Peptides Containing Cysteine Residues

Márton Lukács, Dóra Csilla Pálinkás, Györgyi Szunyog, and Katalin Várnagy*

Table S1 The stability constants of zinc(II), cadmium(II) and lead(II) complexes of peptides containing one cysteinyl residue (T = 298 K, I = 0.2 M)

$\log\beta$	Ac-AAAC-NH_2			Ac-SAAC-NH_2			Ac-CGAA-NH_2		Ac-CGAK-NH_2			Ac-CGAD-NH_2		Ac-CGAH-NH_2		
	Zn(II)	Cd(II)	Pb(II)	Zn(II)	Cd(II)	Pb(II)	Zn(II)	Cd(II)	Zn(II)	Cd(II)	Pb(II)	Zn(II)	Cd(II)	Zn(II)	Cd(II)	Pb(II)
[MHL]	—	—	—	—	—	—	—	—	—	15.6(2)	15.5(1)	—	—	11.6(2)	12.32(3)	12.46(6)
[ML]	—	—	6.2(1)	5.00(8)	—	6.4(1)	—	—	—	—	—	5.3(1)	6.40(5)	—	6.22(6)	6.14(5)
[MH_2L_2]	—	—	—	—	—	—	—	—	29.60(9)	31.46 (9)	31.48(5)	—	—	—	—	—
[MHL_2]	—	—	—	—	—	—	—	—	21.4(2)	22.8(1)	22.2(1)	—	—	18.94(2)	19.41(2)	—
[ML_2]	9.72(4)	11.71(3)	—	9.80(8)	11.72(2)	—	9.79(8)	11.36(5)	—	—	12.00(8)	10.3(1)	11.6(1)	12.44(4)	12.62(3)	11.41(5)
[$MH_{-1}L_2$]	1.3(1)	—	—	2.22(7)	0.97(3)	—	1.6(1)	1.7(1)	1.2(2)	2.0(1)	—	1.7(2)	—	3.57(7)	2.28(7)	—
pK_{11}*	—	—	—	—	—	—	—	—	—	—	—	—	—	—	6.10	6.32
pK_{21}**	—	—	—	—	—	—	—	—	8.2	8.7	9.3	—	—	—	—	—
pK_{22}**	—	—	—	—	—	—	—	—	10.1	10.4	10.2	—	—	6.53	6.79	—
pK_{23}**	8.4	—	—	7.58	10.75	—	8.2	9.7			—	8.6	—	8.87	10.34	—

* $\log\beta$[MHL] – $\log\beta$[ML]

** $\log\beta[MH_xL_2] – \log\beta[MH_{x-1}L_2]$

Table S2 The stability constants of zinc(II), cadmium(II) and lead(II) complexes of peptides containing two cysteinyl residues residue (T = 298 K, I = 0.2 M)

logβ	Ac-SCCS-NH$_2$			Ac-CSC-NH$_2$			Ac-CSSC-NH$_2$			Ac-CSSACS-NH$_2$		
	Zn(II)	Cd(II)	Pb(II)	Zn(II)	Cd(II)	Pb(II)	Zn(II)	Cd(II)	Pb(II)	Zn(II)	Cd(II)	Pb(II)
[ML]	10.05(2)	12.60(3)	10.86(4)	10.11(4)	12.69(2)	12.06(5)	10.47(2)	13.04(2)	11.72(3)	9.16(1)	11.47(3)	10.81(5)
[$MH_{-1}L$]	1.36(6)	2.59(5)	0.6(2)	2.21(5)	2.57(7)	—	2.15(5)	2.38(5)	1.03(5)	0.88(4)	0.83(5)	–0.4(1)
[$MH_{-2}L$]	–8.84(7)	—	—	–8.17(6)	–9.1(1)	—	–8.40(6)	—	—	–9.82(4)	—	—
[ML_2]	16.7(1)	19.1(2)	15.5(2)	19.77(5)	21.0(1)	—	19.2(1)	21.1(2)	—	17.07(8)	18.6(1)	—
pK_1*	8.69	10.01	10.3	7.90	11.67	—	8.32	10.66	10.69	8.28	10.64	11.2
pK_2*	10.20	—	—	10.38	—	—	10.55	—	—	10.70	—	—

* logβ[ML] – logβ[$MH_{-x}L$], x = 1, 2

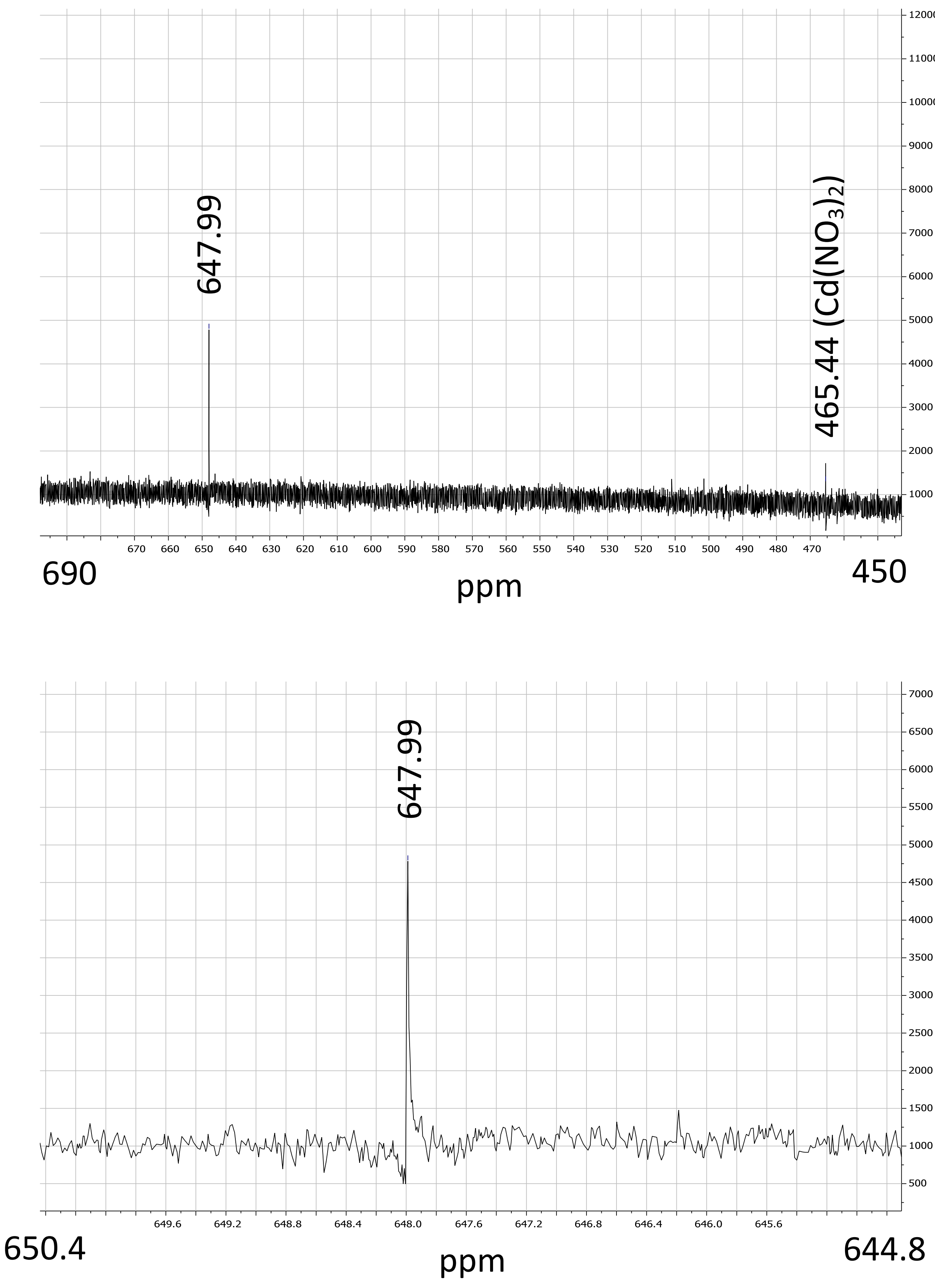

Figure S1: ^{113}Cd spectrum of Cd(II)-Ac-CSSC-NH$_2$ system (in DMSO-d^6)

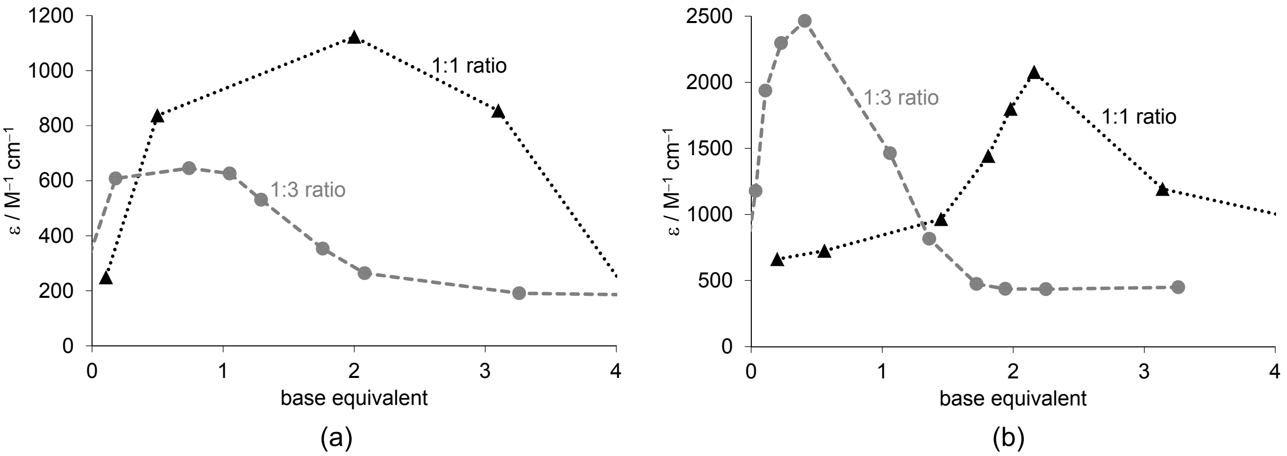

Figure S2: Change of the molar absorptivity of Ni(II):Ac-CSC-NH_2 1:1 and 1:2 solutions at 433 nm (a) and Ni(II):Ac-CSSC-NH_2 1:1 and 1:2 solutions at 419 nm (b) in the function of equivalent of base (c_L = 1 mM)

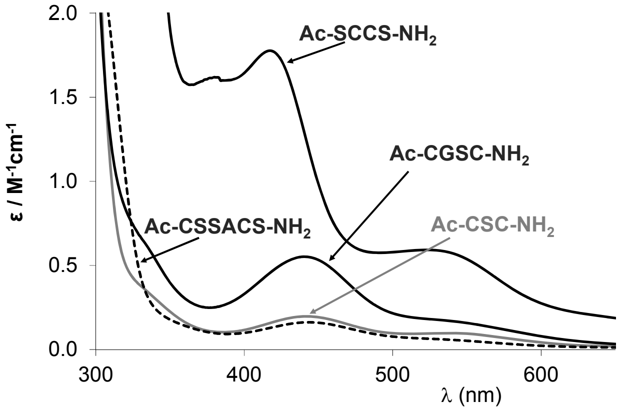

Figure S3: The UV-Vis spectra of [$NiH_{-3}L$] complexes of the peptides containing two cysteinyl residues

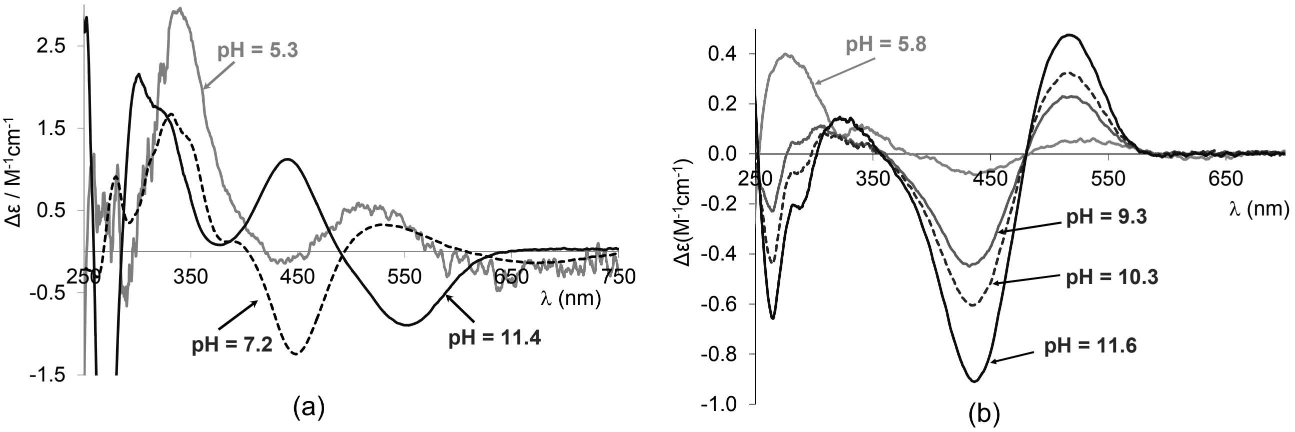

Figure S4: The CD spectra of Ni(II):Ac-CSC-NH_2 1:1 (a) and Ni(II):Ac-CSSC-NH_2 1:1 (b) systems (c_L = 1 mM)
